# *NF-κB (p65, p50)*, *IL-18*, and *IL-10* as Therapeutic Targets in Prostate Cancer and BPH: Molecular Insights into Inflammation-Driven Pathogenesis

**DOI:** 10.31557/APJCP.2026.27.1.381

**Published:** 2026-01-22

**Authors:** Shailendra Dwivedi, Sapna Saini, Ajay, Anjali Krishna NP, Akash Bansal, Ravi Shankar Sharma, Shashank Shekhar, Deepika, Apul Goel, Sanjay Khattri

**Affiliations:** 1 *Department of Biochemistry, All India Institute of Medical Sciences (AIIMS) Gorakhpur, Uttar Pradesh, 273008, India. *; 2 *Department of Anaesthesia, All India Institute of Medical Sciences (AIIMS) Gorakhpur, Uttar Pradesh, 273008, India.*; 3 *Department of Radiotherapy, All India Institute of Medical Sciences (AIIMS) Gorakhpur, Uttar Pradesh, 273008, India. *; 4 *Department of Pathology, All India Institute of Medical Sciences (AIIMS) Gorakhpur, Uttar Pradesh, 273008, India.*; 5 *Department of Urology, King George’s Medical University, Lucknow, Uttar Pradesh 226003, India.*; 6 *Principal, Maharaja Suhel Dev Autonomous State Medical College & Mahrishi Balark Hospitals, Bahraich, Uttar Pradesh 271801, India.*

**Keywords:** Prostate cancer, NF-κB- IL-18- IL-10, biomarkers, BPH, occupational exposure, inflammation

## Abstract

**Background:**

Prostate cancer is a significant global health burden and is the second most commonly diagnosed malignancy among men. Chronic inflammation and environmental exposures, including occupational toxins, are increasingly recognized as key contributors to its development. Nuclear factor kappa B (*NF-κB*) subunits *p65* and p50, along with the cytokines *IL-18* and *IL-10*, are central mediators of inflammation, but remain understudied in the context of benign prostatic hyperplasia (BPH) and occupation-related risks. This study investigates the expression patterns of these markers in prostate cancer, BPH, and healthy individuals, and examines their association with disease stage and occupational exposure

**Methods:**

A total of 664 participants were enrolled, including 285 prostate cancer patients, 94 BPH cases, and 285 healthy controls. Peripheral blood samples were collected and analyzed for mRNA expression using quantitative real-time PCR (qRT-PCR), and for protein levels using ELISA. Statistical comparisons among groups and two-way ANOVA were performed to evaluate the effects of disease status and occupation. Correlation analysis was used to assess the associations between *NF-κB* and cytokine levels.

**Results:**

*NF-κB* *p65* and p50, as well as *IL-18* and *IL-10*, were significantly upregulated in prostate cancer compared to BPH and controls (p < 0.0001). Expression levels increased with cancer stage and metastatic status. Among occupational groups, tannery workers exhibited the highest biomarker expression. Significant positive correlations were found between *NF-κB* subunits and both cytokines. Conclusion: *NF-κB* and its downstream cytokines, *IL-18* and *IL-10*, may serve as inflammation-driven, noninvasive biomarkers for prostate cancer diagnosis, staging, and risk stratification, particularly in populations exposed to environmental factors.

## Introduction

Prostate cancer is the second most frequently diagnosed malignancy and a major cause of cancer-related mortality among men worldwide [1]. Global epidemiological trends reveal rising incidence in low- and middle-income countries, contrasting with declining mortality in high-income regions due to enhanced screening and treatment strategies [2]. This disparity reflects unequal access to healthcare, early detection, and effective interventions, particularly in rapidly transitioning economies [3]. Ethnic and geographical differences in prostate cancer incidence and outcomes are further influenced by genetic predisposition and healthcare inequalities [4].

Although prostate-specific antigen (PSA) testing remains a cornerstone of prostate cancer screening, its limited specificity often results in false positives, overdiagnosis, and unnecessary treatments [5]. Including benign prostatic hyperplasia (BPH) as a comparative group in molecular investigations may help differentiate inflammation-related markers and improve diagnostic specificity [6]. With projections estimating over 2.2 million prostate cancer cases globally by 2040, early detection and risk stratification remain urgent public health priorities [4].

The etiology of prostate cancer is multifactorial, involving complex interactions among genetic susceptibility, androgen signaling, environmental exposures, and chronic inflammation [7]. Inflammatory processes play a critical role in initiating and sustaining carcinogenesis by promoting oxidative stress, DNA damage, angiogenesis, and immune evasion [8]. Variations in genes encoding inflammatory mediators, including *COX-2, IL1B, IL6, IL8, IL10, TNF*, and *TLR4*, have been linked to increased prostate cancer risk and disease aggressiveness [9]. Additionally, epigenetic modifications contribute significantly to tumor development and progression [10].

Central to inflammatory regulation is the transcription factor complex *NF-κB*, comprising key subunits *p65* (RelA) and *p50*, which translocate to the nucleus upon activation by cytokines, microbial agents, or oxidative stress [11]. Once activated, *NF-κB* promotes the transcription of genes involved in cell survival, angiogenesis, immune modulation, and tumor progression [12]. Aberrant or constitutive activation of *NF-κB* has been implicated in treatment resistance, advanced disease stages, and castration-resistant prostate cancer [13].

Downstream of *NF-κB* signaling are cytokines such as interleukin-18 (*IL-18*) and interleukin-10 (*IL-10*), which play opposing but complementary roles in tumorigenesis. *IL-18* amplifies Th1 responses and induces interferon-gamma, sustaining a chronic inflammatory milieu conducive to tumor growth [14]. In contrast, *IL-10* exerts immunosuppressive effects by downregulating antigen presentation and inhibiting T-cell activation, thereby promoting immune escape in prostate cancer [15]. The dynamic interplay between *IL-18* and *IL-10*, both regulated by *NF-κB*, reflects a complex immunological landscape that supports tumor development and progression [16].

Promoter polymorphisms in *IL-18* and *IL-10* genes influence cytokine expression and are linked to survival outcomes in prostate cancer patients [17]. Elevated serum levels of *IL-18* and *IL-10* have been shown to have diagnostic and prognostic importance in advanced disease, highlighting their value as inflammation-related biomarkers [18]. Additionally, *NF-κB* signaling supports the maintenance of cancer stem cells and interacts with folate metabolism pathways, further contributing to tumor development and resistance mechanisms [19].

The clinical overlap between BPH and prostate cancer poses diagnostic challenges. Investigating *NF-κB* and its downstream cytokines in both conditions may reveal molecular signatures that help differentiate benign from malignant pathologies [20]. Blood-based inflammatory biomarkers, if validated, offer a non-invasive approach to early detection and disease monitoring [21].

Environmental and occupational exposures also contribute to prostate cancer risk. Workers in high-risk industries such as tanning, agriculture, and ordnance are exposed to carcinogens like cadmium, arsenic, and organochlorine compounds, which may trigger oxidative stress and *NF-κB* activation [22]. Meta-analyses confirm increased prostate cancer risk associated with such exposures, particularly to organochlorine pesticides and heavy metals [23]. The interaction between inflammation and occupational hazards may amplify cancer risk in susceptible individuals [24].

Vitamin D deficiency further exacerbates inflammation by activating *NF-κB*, while tobacco exposure alters cytokine profiles, enhancing inflammatory responses and immune suppression in prostate cancer [25, 26] These findings emphasize the need for comprehensive molecular profiling that incorporates environmental and lifestyle risk factors.

Given this context, the present study examines the expression of *NF-κB* subunits *p65* and *p50*, along with *IL-18* and *IL-10*, in patients with prostate cancer, BPH, and healthy controls. It further evaluates the influence of occupational exposures and cancer stage on these biomarkers, aiming to identify non-invasive, inflammation-driven molecular indicators for diagnosis, staging, and potential therapeutic targeting in prostate cancer.

## Materials and Methods

### Study Design and Population

This case-control observational study included 664 male participants aged 50–85 years, recruited from a tertiary care urology clinic. The study population comprised three groups: histopathologically confirmed prostate cancer patients (n=285), benign prostatic hyperplasia (BPH) cases (n=94), and age-matched healthy male controls (n=285) with no history of prostate disease. Diagnoses were established through digital rectal examination, serum PSA levels, and prostate biopsy. Healthy controls were selected during routine health screenings and confirmed to have normal PSA levels and no clinical abnormalities.

All participants completed a structured questionnaire that captured demographic characteristics, occupational history, lifestyle factors (e.g., smoking, alcohol consumption), and comorbidities. Written informed consent was obtained, and the Institutional Human Ethics Committee approved the study protocol in accordance with the Declaration of Helsinki.

### Assessment of Occupational Exposure

Occupational exposure was assessed using a validated questionnaire that detailed past and current employment in high-risk industries, including agriculture, tanning, and ordnance sectors. Exposure classification was based on predefined profiles involving contact with known carcinogens such as cadmium and organochlorine compounds.

### Sample Collection and Molecular Analyses

Peripheral venous blood (5 mL) was collected in EDTA tubes for RNA analysis and in serum separator tubes for ELISA. Samples were processed within two hours. Peripheral blood mononuclear cells (PBMCs) were isolated using Ficoll-Paque density gradient centrifugation. Total RNA was extracted using TRIzol reagent (Invitrogen) and quantified spectrophotometrically.

Complementary DNA (cDNA) was synthesized from 1 µg of RNA using a commercial reverse transcription kit (Thermo Fisher Scientific). Quantitative real-time PCR (qRT-PCR) was performed using SYBR Green chemistry on a Bio-Rad CFX96 system. Specific primers for *NF-κB* subunits (*p65*, *p50*), *IL-18*, *IL-10*, and *GAPDH* (housekeeping gene) were designed using Primer-BLAST and validated for specificity. Amplification involved initial denaturation at 95°C for 10 min, followed by 40 cycles of 95 °C for 15 s and 60 °C for 1 min. Each sample was analyzed in triplicate. Relative gene expression was calculated using the ΔΔCt method.

### Elisa For Protein Quantification

Serum protein levels of *NF-κB* *p65* and *p50* were measured using high-sensitivity ELISA kits (Abcam, UK), while *IL-18* and *IL-10* were quantified using kits from R&D Systems. All assays were performed in duplicate according to the manufacturers’ instructions. Absorbance was measured at 450 nm, and concentrations were calculated from standard curves.

### Statistical Analysis

Data analysis was conducted using SPSS version 26.0 and GraphPad Prism version 9.0. Normality was assessed with the Shapiro-Wilk test. Continuous variables are expressed as mean ± standard deviation (SD) or median (IQR), as appropriate. Group comparisons were conducted using one-way ANOVA with Tukey’s post hoc test or Kruskal-Wallis test with Dunn’s correction, depending on data distribution. A two-way ANOVA was used to assess interaction effects between the disease group and occupational exposure.

Pearson’s correlation was applied to assess the associations between *NF-κB* and cytokine levels, following the assumptions of linearity and the absence of outliers. All tests were two-tailed with significance set at p < 0.05. Power analysis confirmed greater than 90% power to detect moderate effect sizes across groups.

## Results

### NF-κB and cytokine expression in Prostate cancer, BPH, and Control Groups

Table 1 presents a comparative analysis of *NF-κB* subunits (*p65* and *p50*) at both mRNA and protein levels across prostate cancer, BPH, and healthy controls. A statistically significant upregulation of *NF-κB* *p65* and *p50* was observed in prostate cancer patients compared to BPH and controls (p<0.0001 for both mRNA expression and protein levels). Relative mRNA expression of *NF-κB* *p65* was markedly elevated in prostate cancer (18.57±12.66) compared to BPH (2.59±2.51) and controls (1.98±1.84). A similar pattern was seen for *p50* (11.57±8.31 in cancer vs. 2.02±2.09 in BPH and 1.79±1.63 in controls). ELISA measurements further confirmed this trend, with significantly higher serum protein levels of *p65* and *p50* in cancer patients (375.38±105.84 pg/mL and 150.79±35.33 pg/mL, respectively) compared to the BPH and control groups. *GAPDH* Ct values showed no significant difference among groups (p=0.94), validating normalization. These findings suggest robust transcriptional and translational activation of *NF-κB* in prostate malignancy.

### Occupation-wise Expression Patterns of NF-κB Markers

As shown in Table 2, subgroup analysis based on occupational exposure revealed that tannery workers exhibited the highest levels of *NF-κB* *p65* and *p50* expression across all clinical groups. Among prostate cancer patients, ELISA-derived protein levels of *p65* reached 408.68±105.20 pg/mL in tanners, compared to 386.25±104.47 pg/mL in ordnance workers and 362.42±97.71 pg/mL in sedentary individuals. Correspondingly, *p50* levels were also elevated in tanners (154.80±35.97 pg/mL), followed by agricultural workers and ordnance-exposed individuals. Although the group effect was statistically significant (p<0.0001), the occupation effect was marginal (p=0.08), and the interaction term approached significance (p=0.09), suggesting potential synergistic effects between disease status and environmental exposure. These occupational patterns as shown in Figure 1A and Figure 1B reinforce the inflammatory risk posed by industrial toxins, particularly chromium compounds and aromatic amines.

### NF-κB and Cytokine Expression by TNM Stage and Metastasis

Table 3 illustrates the expression trends of *NF-κB* *p65*/*p50*, *IL-18*, and *IL-10* across different TNM stages among prostate cancer patients. Progressive disease stages were associated with a significant increase in mRNA expression and a reduction in Ct values of both *NF-κB* subunits. Relative expression of *p65* rose from 12.51±10.47 (T1) to 41.19±34.93 (M1), while *p50* expression increased from 5.57±5.26 to 34.44±38.10 (p<0.0001 for both). Protein levels of *p50* increased significantly with disease advancement (p=0.001), while *p65* protein plateaued beyond T2 (p=0.21), indicating potential post-transcriptional regulation.

Similarly, the expression of *IL-18* and *IL-10* showed a stage-wise escalation. Relative *IL-18* mRNA expression rose from 1.06±0.97 in T1 to 14.51±10.24 in metastatic (M1) cases (p=0.03), while *IL-10* increased from 1.18±3.23 to 15.60±7.56 (p<0.0001). ELISA-based serum *IL-18* levels also showed a significant increase across stages (p=0.008), whereas *IL-10* protein levels did not significantly differ (p=0.26). PSA levels showed an upward trend with disease progression but did not reach statistical significance (p=0.23). These results suggest that *IL-18* and *IL-10* are transcriptionally responsive to disease severity and may act downstream of *NF-κB* activation, contributing to inflammation-mediated tumor progression.

## Discussion

This study highlights the critical role of *NF-κB* signaling in prostate cancer progression, evidenced by the consistent overexpression of its subunits *p65* and *p50* in cancer patients relative to BPH and healthy controls. Notably, while both subunits were elevated at the mRNA and protein levels, *p50* exhibited a stronger correlation with disease stage, suggesting differential post-transcriptional regulation [27]. These observations align with *NF-κB*’s known function in promoting tumorigenesis through proliferation, survival, and immune modulation [28].

While *p65* protein expression plateaued in advanced stages, *p50* levels continued to rise, supporting its potential utility as a stage-specific prognostic marker [29]. Persistent *NF-κB* activation is implicated in tumor progression and treatment resistance via the upregulation of angiogenic and metastatic genes such as IL-8, VEGF, and MMPs [30] . Disrupting this pathway may sensitize prostate cancer cells to radiotherapy and chemotherapy [31, 32]

*IL-18* and *IL-10*, although immunologically divergent, were both significantly upregulated as disease progression advanced. *IL-18*, a pro-inflammatory cytokine, enhances NK cell and Th1 responses, but excessive levels may sustain chronic inflammation and tumor growth [33]. Conversely, *IL-10* suppresses T-cell activity and antigen presentation, enabling immune evasion [34]. Despite opposing immune functions, both cytokines contribute to a tumor-supportive microenvironment, mirroring *NF-κB* expression patterns [35]. Their upregulation alongside *NF-κB* highlights a coordinated inflammatory and immunosuppressive response in prostate cancer [36].

Our findings further emphasize the influence of occupational exposure. Among high-risk professions, tannery workers showed the highest biomarker levels, likely due to chronic exposure to genotoxic agents such as chromium and aromatic amines [37]. Two-way ANOVA revealed a borderline significant interaction between occupation and disease, suggesting a synergistic effect on inflammation-driven oncogenesis. These results align with existing literature on occupational carcinogens and *NF-κB* activation [38].

Additionally, elevated cytokine levels among tobacco users, including those consuming smokeless forms, support a link between exogenous inflammatory triggers and prostate tumorigenesis [39]. Interestingly, previous cohort studies suggest that *IL-10* may have dual roles, protective or tumor-promoting, depending on the immune context [40]. Our findings support the context-dependent involvement of this mechanism in the progression of cancer.

The therapeutic relevance of these markers is promising. High-dose *IL-10* formulations (e.g.., pegilodecakin) have shown promise in restoring CD8⁺ T-cell responses and enhancing the efficacy of checkpoint inhibitors. Similarly, *IL-18* is being explored in vaccine and CAR-T cell platforms due to its immunostimulatory profile [41]. These developments underscore the translational potential of *IL-10* and *IL-18* as therapeutic targets for immunotherapy.

However, discordance between transcript and protein levels, particularly for *p65*points to possible post-transcriptional regulation or saturation in protein release, highlighting the necessity of integrating transcriptomic and proteomic data for biomarker validation [42]. Multi-omics approaches may better capture the complex molecular landscape of prostate cancer, facilitating precision oncology.

Given the robust association of *NF-κB* with tumor progression and environmental exposure, trials of *NF-κB* inhibitors in inflammation-driven cancers may be extended to prostate cancer [43]. Modulating cytokine signaling could also complement standard therapies [44]. Nevertheless, limitations of this study include its cross-sectional design and reliance on blood-based markers, which may not fully reflect tumor-specific events. Future longitudinal studies integrating tissue-level analyses are warranted.

In summary, *NF-κB* *p65*/*p50*, *IL-18*, and *IL-10* are significantly overexpressed in prostate cancer, correlating with disease stage and occupational exposure. These biomarkers may facilitate non-invasive diagnosis, risk stratification, and therapeutic targeting, especially in populations exposed to environmental toxins. Incorporating these findings into clinical practice and occupational health policies could improve early detection and outcomes. Prospective validation is essential to confirm their utility in precision medicine.

**Table 1:** Comparison of *NF-κB p65* and *p50* Expression at mRNA and Protein Levels in BPH, Prostate Cancer, and Control Groups

Biomarker	Parameter	BPH (n=94)	Prostate Cancer (n=285)	Control (n=285)	p-value
*NF-κB p65*	Ct value (mean ± SD)	24.10 ± 0.94	21.02 ± 1.11^12^	24.45 ± 1.04	0.01*
	Relative mRNA expression	2.59 ± 2.51^1^	18.57 ± 12.66^12^	1.98 ± 1.84^2^	<0.0001*
	ELISA protein (pg/mL)	60.42 ± 6.78^1^	375.38 ± 105.84^12^	39.65 ± 39.64^2^	<0.0001*
*NF-κB p50*	Ct value (mean ± SD)	24.47 ± 0.90	21.80 ± 1.21^12^	24.50 ± 0.75	0.01*
	Relative mRNA expression	2.02 ± 2.09¹	11.57 ± 8.31^12^	1.79 ± 1.63^2^	<0.0001*
	ELISA protein (pg/mL)	25.49 ± 4.67^1^	150.79 ± 35.33^12^	23.40 ± 5.51^2^	<0.0001*
*GAPDH*	Ct value (mean ± SD)	24.98 ± 0.68	24.95 ± 0.66	24.95 ± 0.65	0.94

^12^, Significantly different from both BPH and control (Tukey’s multiple comparisons, p < 0.05); ^1^, Significantly different from cancer (p < 0.05); ^2^, Significantly different from cancer (p < 0.05); *, Statistically significant at α = 0.05 (ANOVA or Kruskal-Wallis as appropriate)

**Figure 1A:**
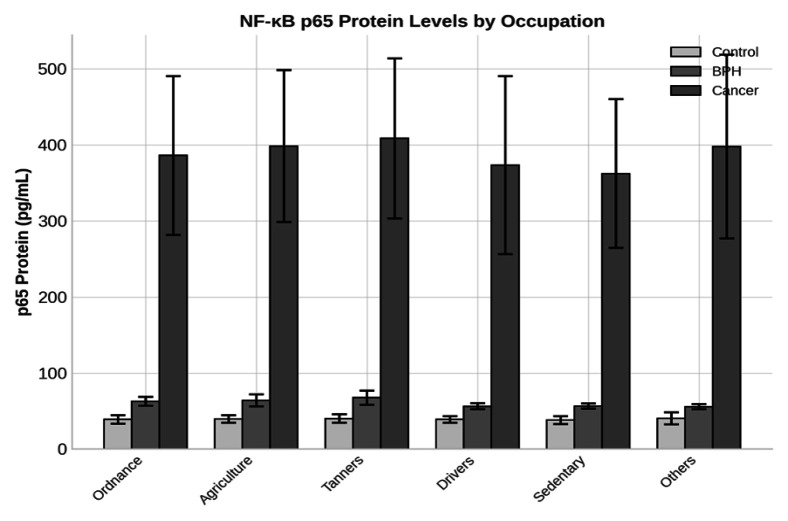
*NF-κB p65* Protein Levels by Occupation. Mean ± SD of p65 protein (pg/mL) in controls, BPH, and cancer across occupations. Two-way ANOVA: group effect significant (p < 0.0001); occupation effect marginal (p ≈ 0.08); interaction p ≈ 0.09.

**Figure 1B:**
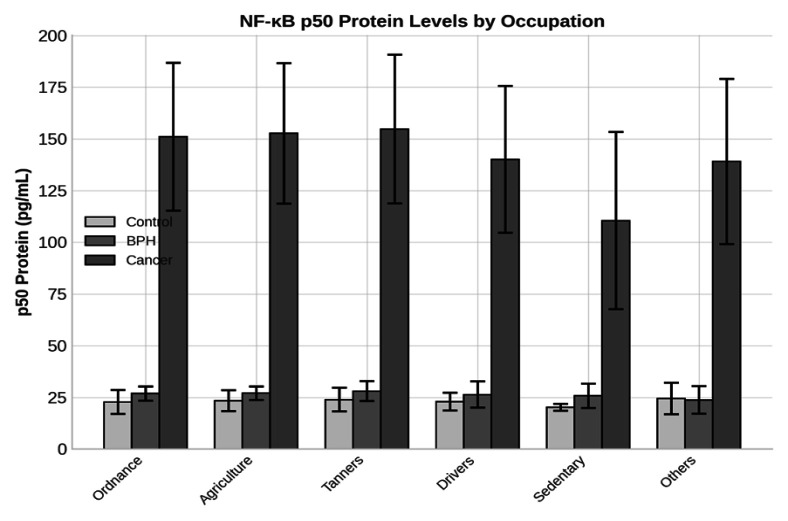
*NF-κB p50 *Protein Levels by Occupation. Mean ± SD of p50 protein (pg/mL) in controls, BPH, and cancer across occupations. Two-way ANOVA: group effect significant (p < 0.0001); occupation effect not significant.

**Table 2:** *NF-κB* and Cytokine Expression Across Occupations

Biomarker / Group	Ordnance	Agriculture	Tanners	Drivers	Sedentary	Others
Ct *NF-κB p65* (BPH)	24.48±0.79	23.73±0.93	24.24±0.79	23.54±0.72	24.72±1.35	23.62±0.99
Ct *NF-κB p65* (Cancer)	21.05±1.09	20.96±1.08	21.14±1.04	21.15±1.51	20.59±0.76	21.09±1.08
Ct *NF-κB p65* (Control)	24.28±1.15	24.57±0.97	24.45±0.99	24.20±1.11	24.88±1.39	24.76±0.97
Ct *NF-κB p50* (BPH)	24.24±0.70	24.45±0.96	25.56±1.12	24.38±0.87	24.78±0.79	24.82±0.70
Ct *NF-κB p50* (Cancer)	21.80±1.24	21.63±1.23	22.34±2.56	22.01±1.20	21.10±0.83	21.19±1.16
Ct *NF-κB p50* (Control)	24.49±0.76	24.55±0.74	24.81±1.08	24.49±0.91	24.71±0.86	24.81±0.66
mRNA *p65* (BPH)	1.86±1.90	2.68±1.98	2.15±1.50	1.99±0.58	1.32±0.99	2.11±0.96
mRNA *p65 *(Cancer)	18.86±14.38	17.95±9.26	19.45±9.82	17.94±17.77	16.79±6.63	17.64±3.26
mRNA *p65* (Control)	2.43±2.54	1.69±1.43	2.77±2.26	2.63±1.98	2.46±2.07	1.45±1.38
mRNA *p50* (BPH)	2.30±2.34	2.95±2.81	3.83±2.47	2.52±1.63	1.06±0.46	1.36±0.81
mRNA* p50* (Cancer)	11.59±8.19	12.76±9.21	11.83±8.19	10.81±7.07	9.85±3.43	10.67±2.11
mRNA *p50* (Control)	1.86±1.73	1.71±1.57	2.49±2.14	1.87±1.33	2.42±1.48	0.99±0.27
ELISA *p65* (BPH)	62.93±5.83	64.13±7.79	67.73±9.32	56.39±3.88	56.78±3.32	55.77±3.16
ELISA *p65* (Cancer)	386.25±104.47	398.59±99.81	408.68±105.20	373.46±117.03	362.42±97.71	398.02±120.87
ELISA *p65* (Control)	39.02±5.71	39.62±5.09	40.19±5.79	39.02±4.39	38.22±5.28	40.51±7.86
ELISA *p50* (BPH)	26.86±3.42	27.03±3.25	28.02±4.81	26.33±6.35	25.78±5.92	23.75±6.64
ELISA *p50* (Cancer)	151.10±35.73	152.69±33.96	154.80±35.97	140.12±35.46	110.51±42.83	139.08±39.90
ELISA *p50* (Control)	22.83±5.77	23.38±5.08	23.94±5.79	22.95±4.35	20.22±1.72	24.44±7.62

Group effect was significant (p < 0.0001), occupational effect marginal (p = 0.08), and group × occupation interaction borderline (p = 0.09); cancer vs. control was significant (p < 0.0001), BPH vs. control was not (p = 0.09).

**Table 3:** Comparison of Biochemical, Immunological, and Expression by TNM Classification among Cancer Patients

Parameters	TNM classification						p-value
	T1	T2	T3	T4	N	M	
PSA level	19.70±7.24	23.55±8.39	29.61±21.73	34.31±27.73	34.45±15.31	34.52±25.62	0.23
Interileukin-18 (pg/ml)	248.65±28.80	260.19±21.87	262.02±21.01	266.80±22.10	266.94±30.90	273.47±20.19	0.008*
Interleukin-10 (pg/ml)	11.81±2.89	11.94±1.85	11.89±1.97	12.16±1.80	12.51±3.19	12.90±1.63	0.26
Ct value *Nf-Kb*	21.42±1.58	21.18±1.24	21.09±0.94	21.07±1.16	20.54±1.09	19.95±1.04	0.0001*
* p65* mRNA							
Ct value Nf-kb	22.40±1.04	22.24±1.08	22.19±1.19	21.88±1.30	20.89±1.84	20.78±1.90	0.001*
p50 mRNA							
Ct value of *GAPDH*	24.40±0.53	24.70±0.56	24.89±0.64	24.88±0.73	24.90±0.85	24.84±0.68	0.08
Relative mRNA expression *p65*	12.51±10.47	16.04±14.86	17.64±14.02	20.02±22.07	26.74±22.95	41.19±34.93	0.0001*
Relative mRNA expression *p50*	5.57±5.26	7.40±5.66	10.34±13.05	11.73±12.21	27.57±30.79	34.44±38.10	0.0001*
ELISA-*P65*	346.95±119.30	363.14±113.99	366.11±100.99	383.62±101.89	404.07±90.51	409.13±87.18	0.21
ELISA *P50*	140.10±31.05	143.54±34.62	143.90±31.92	149.08±37.22	172.23±33.79	184.24±30.36	0.001*
mRNA (Ct) of *IL-18*	25.16±2.14	24.59±3.08	24.18±3.13	23.97±3.61	23.82±2.86	22.33±1.79	0.04*
mRNA (Ct) of* IL-10*	27.04±2.97	26.62±2.62	26.44±2.32	26.22±2.73	25.26±2.62	24.93±2.23	0.04*
Relative mRNA expression *IL-18*	1.06±0.97	6.49±13.60	9.13±15.01	13.74±24.83	13.97±6.97	14.51±10.24	0.03*
Relative mRNA expression *IL-10*	1.18±3.23	2.67±7.22	9.42±11.66	12.54±15.45	15.47±10.89	15.60±7.56	0.0001*

## Author Contribution Statement

All authors have read and approved the final manuscript and consent to its publication. Informed consent was obtained from all participants for inclusion and publication of anonymized data.
